# Supplementary figures and images for: Network feature-based phenotyping of leaf venation robustly reconstructs the latent space
Source: PLoS Comput Biol. 2023 Jul 20;19(7):e1010581. doi: 10.1371/journal.pcbi.1010581 (PMC10358950; doi:10.1371/journal.pcbi.1010581)

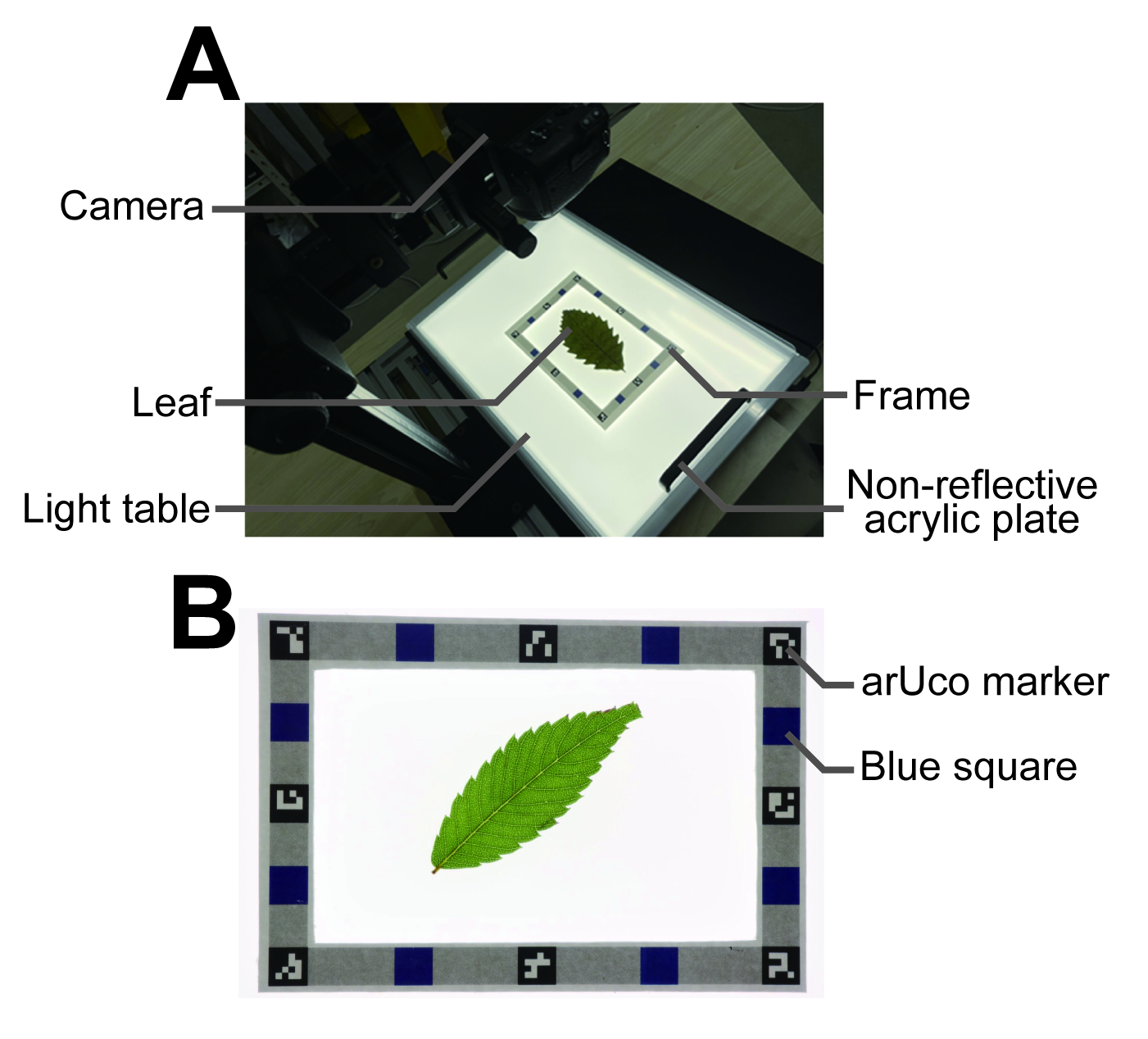

Supplement: S1 Fig — (A) A sampled leaf was placed on a light table and in a frame for correction and was covered with a non-reflective acrylic plate. Leaves were captured using a digital camera. (B) The frame had two markers: arUco and blue square markers. The arUco markers were generated using OpenCV to correct for the image angle and distortion. Blue square markers were used to calculate the leaf area. The frame can be adjusted to any size. (TIF) [file pcbi.1010581.s001.tif]

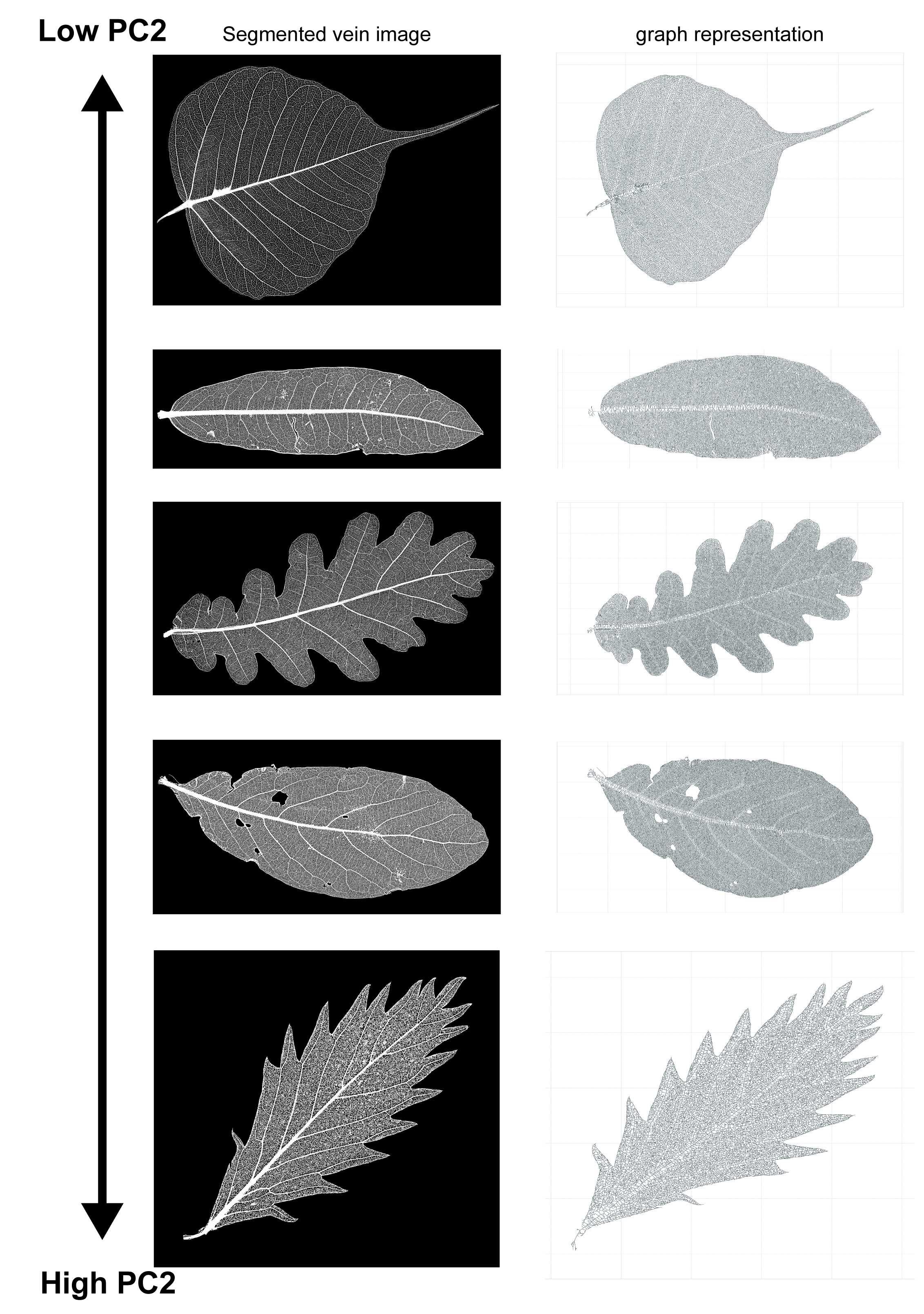

Supplement: S2 Fig — (TIF) [file pcbi.1010581.s002.tif]

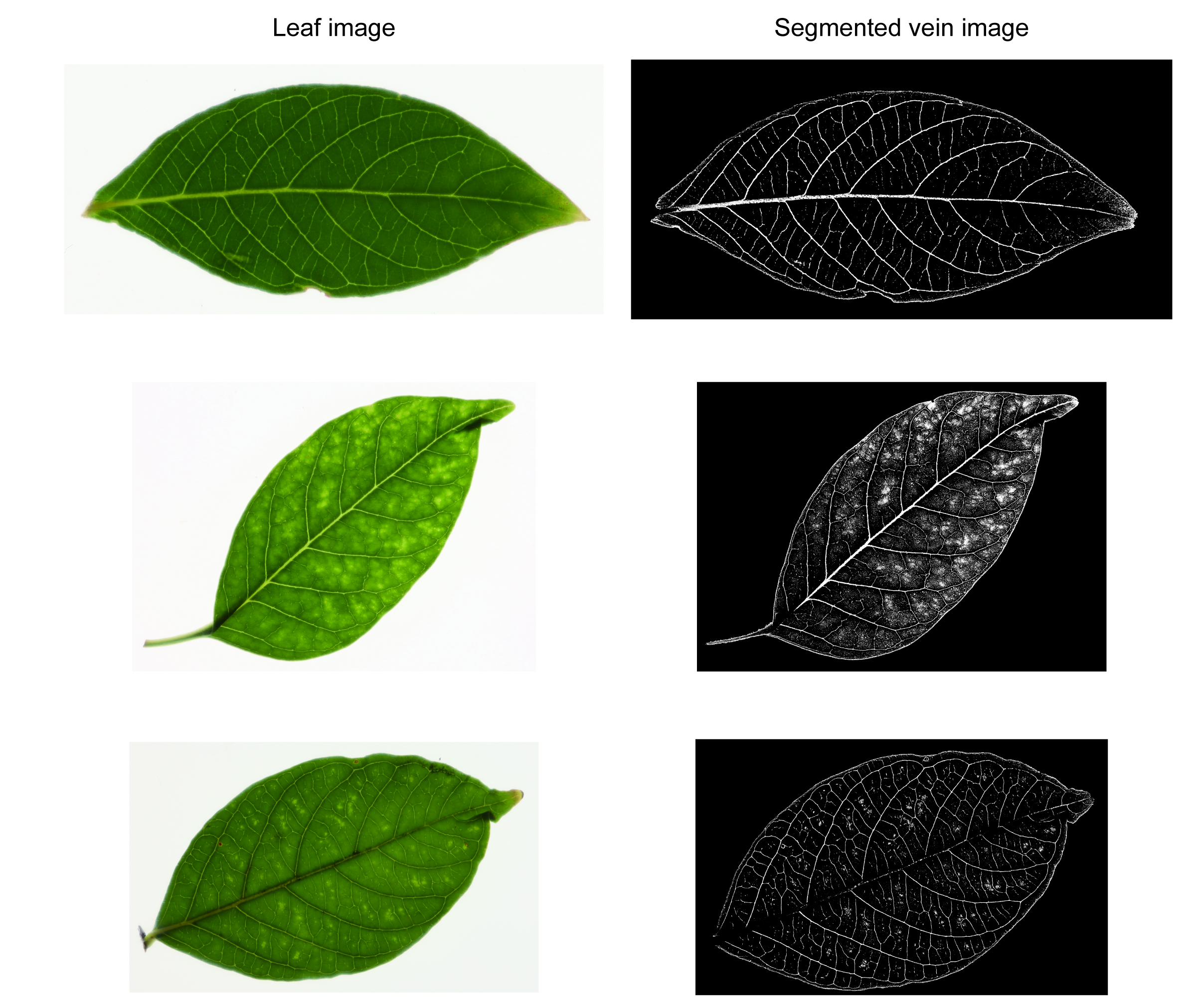

Supplement: S3 Fig — (TIF) [file pcbi.1010581.s003.tif]

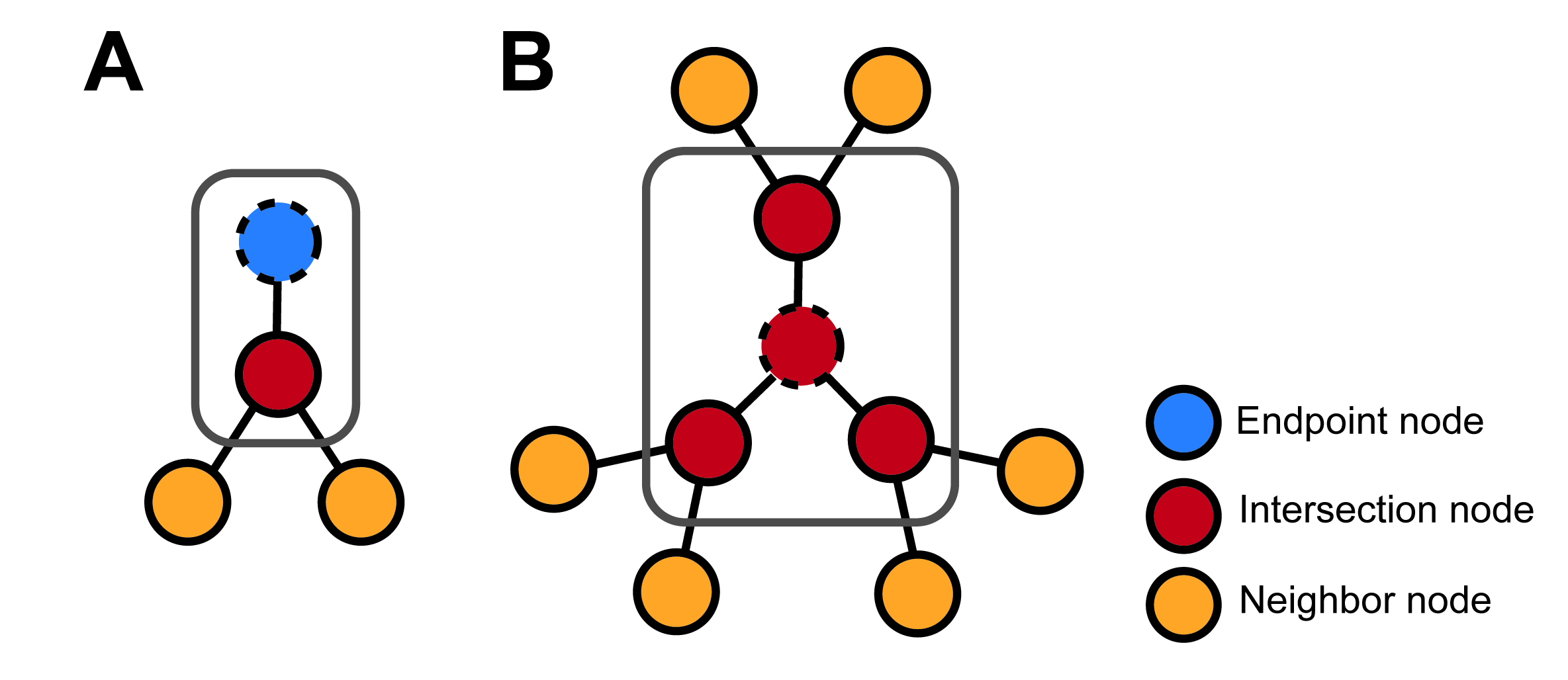

Supplement: S4 Fig — The blue nodes represent endpoint nodes with a degree of one, while the red nodes represent intersection nodes with a degree of three. Nodes with wavy outlines denote the reference node of an egonet, and orange nodes represent neighboring nodes within a one-hop egonet. (A) illustrated an egonet pattern with a neighborhood size of 2, which was relatively prevalent when the reference node was an endpoint node. (B) portrayed another egonet pattern with a neighborhood size of 6, commonly observed when the reference node was an intersection node. (TIF) [file pcbi.1010581.s004.tif]

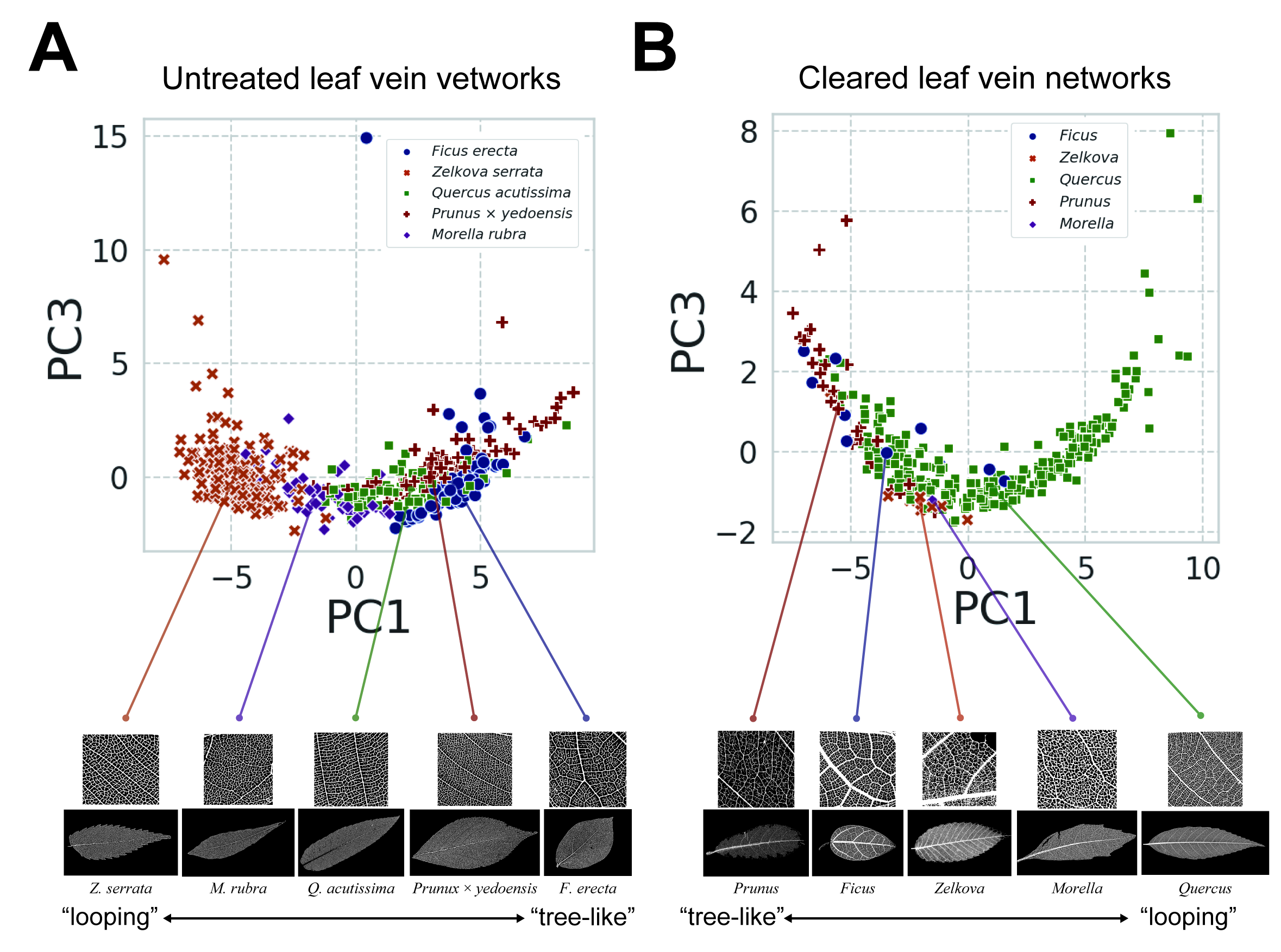

Supplement: S5 Fig — The representative leaf veins for each clade were adopted as the nearest neighbors of the mean of PC scores within the PC1-PC3 space. (A) represented untreated leaves, while (B) represented cleared leaves. (TIF) [file pcbi.1010581.s005.tif]
