# Supplementary material for: Network feature-based phenotyping of leaf venation robustly reconstructs the latent space
Source: PLoS Comput Biol. 2023 Jul 20;19(7):e1010581. doi: 10.1371/journal.pcbi.1010581 (PMC10358950; doi:10.1371/journal.pcbi.1010581)
Supplement: Attachment — Submitted filename: Review_ST.docx [file pcbi.1010581.s006.docx]

Comments for Author

This manuscript reports the characterization of leaf venation patterns, emphasizing the importance of high-throughput and objective statistics using network features. It is pointed out that the conventional analyses of leaf venation were relatively simple and did not capture inherent complexity in venation patterns, therefore, the authors focused on the quantification of vein networks and found a characteristic one-dimensional axis in morphospace which may be a useful morphological descriptor suitable for classifying the leaf venation.

I find the method used in this work fascinating and the findings are interesting and informative, especially the introduction of machine-learning image analysis and the evaluation of the network features. I would say that this work merits the attention of a broad readership and worthy of publication. However, I would like to recommend further clarifications on some minor points outlined below.

Minor points:

1. In the beginning of Abstract (and Author Summary), the purpose of the study seems lacking. There are some key words such as “network pattern is shared in clades”, “functional demands constrain the network structure”, “biased distribution in the morphospace” but then what is the purpose of this study? Possibly, it might be an evolutionary process, species identification only by leaf venation, but it should be clearly stated.
2. In the end of Abstract (and Author Summary), the meanings of functional trade-offs morphological constraints were not clear. I think at least in Author Summary, it should be clearly stated for non-technical readers, e.g., trade-offs between what and what.
3. I felt a bit confused about Abstract and Author Summary because the authors concluded the approach is promising for analyses of the functional and structural properties of veins in Abstract but they concluded the approach is for analyses in various other fields of research including biomimetics, generative design. It could be better to make them consistent.
4. Line 65: It needs a clear explanation about “Leaf venation is under morphological constraints related to functional demands”. I think some key words for function are needed such as hydraulic transporation, transpiration, photosynthesis otherwise the readers may be lost about what the authors want to claim.
5. Line 234 underestimated: I didn’t think there may be a correlation between number of nodes and leaf area because their relationship is not so trivial. Regarding this, the five times difference is not surprising. It might be better to have an expectation or a logical connection before the explanation of Fig.5.
6. Line 257: “PC1 represented the loopiness of higher-order veins” is not trivial. It might be better to state the logical connection between PC1 and the loopiness. I admit that there are some statements in Methods part but it should be also emphasized in the main text. By the way, it should be better to define the loopiness more clearly in the main text. Same for kurtosis and PC3 in Line 273-275.
7. Line 262-265 “The number of … intersection nodes (Fig 7C, high PC1)”: I could not find a logical connection between the number six and intersection nodes. It is better to explain an expectation or possibilities to think about the number of neighbors in the egonet.
8. Line 329-332: This statement is interesting but it seems difficult to follow only from Fig. 7B. It could be better to show a schematic illustration of what the authors found about the network features that retain clade-specific morphological properties. This may enhance impact of this study.
9. Line 332-334: This is also a very interesting discussion but it seems some logical gaps are there. It was difficult to understand “there are structural and functional constraints” only from the one-dimensional occupation pattern. I guessed the network structures are systematically different along the one-dimensional axis and these different structures may contain different function. It could be better to explain such a logical connection between one-dimensional pattern and structural/functional constraint.
10. Line 335-338: This is a view point for the study of leaf venation pattern, and shaped this study. However, I think it was a bit insufficient to explain the finding with some technical terminology such as locally adaptive fluctuating load and one-dimensional Pareto front. I also think this point is fully discussed in the other section besides “the robust reconstruction of morphospace”. Also, up to here, the trade-off between optimizing transport efficiency, construction cost, and robustness to damage is not clear so that it might be better to explain each step by step. Lastly, it seems interesting about the consideration of “along which the network topology changes from a reticulate to a tree-like structure” with viewing fig 2 in ref 13. But the mechanism behind was not clear only from this statement. As it is a very informative discussion, it might be better to have a schematic illustration of the Fig2 in ref 13 like the comment 14 above and precisely explain what the authors want to claim.

Terms and typos:

1. Line 52:the meaning of “(characterized by) relative vein widths” was not clear.
2. Line 57:“improves” hydraulic conductivity, transpiration efficiency, and tolerance to damage relative to what?
3. Line 66: it is better to more clearly define or introduce morphospace. The term “theoretically possible morphological spectrum” was still vague to understand morphospace.
4. Line 88 unsuitable: In the results, it includes raw leaf samples so it might be confusing if the unsuitability is emphasized.
5. Line 93: it might be “a raw leaf…system or cleaned image”
6. Line 99: Loopiness
7. Line 100: It could be better to precisely state the meaning of latent data space.
8. Fig.1 caption: It could be better to define empirical morphospace. This is used for the first time here. The term is explained in the method Morphospace analysis of the leaf vein network but I could not get the meaning of “empirical”.
9. Line 170: Where is the boundary of the leaf vein?
10. Line 216: It might be better to state the machine spec information in the method section.
11. Line 228: I could not get the meaning of “generating one pixel”.
12. Line 248: empirical → data-driven? I want to know the meaning of empirical. Are there any different types of morphospace besides the empirical one.
13. Line 257: I could not get the meaning of “opposite orders for the correspondence of the PC1 axis”
14. Line 335: Theoretically
