# Supplementary material for: Network feature-based phenotyping of leaf venation robustly reconstructs the latent space
Source: PLoS Comput Biol. 2023 Jul 20;19(7):e1010581. doi: 10.1371/journal.pcbi.1010581 (PMC10358950; doi:10.1371/journal.pcbi.1010581)
Supplement: Attachment — Submitted filename: PBP.docx [file pcbi.1010581.s007.docx]

**Response to the reviewers’ comments**

Kohei Iwamasa, Koji Noshita

May 2, 2023

We would like to thank the reviewers for their though reading of the manuscript and their thoughtful comments and suggestions. In this letter, we present the reviewers’ comments in black and provide our responses in red. All changes made in addressing the reviewers’ comments have been incorporated in the revised version of the manuscript, and we believe that the manuscript has been improved by these changes. All coauthors agree with this correction.

**Editor Remarks to Author:**

Thank you very much for submitting your manuscript "Network feature-based phenotyping of leaf venation robustly reconstructs the latent space" for consideration at PLOS Computational Biology.

As with all papers reviewed by the journal, your manuscript was reviewed by members of the editorial board and by several independent reviewers. In light of the reviews (below this email), we would like to invite the resubmission of a significantly-revised version that takes into account the reviewers&apos; comments.

**Reply to Editor Remarks:**

Thank you for giving us an opportunity to revise our manuscript. We have addressed all of the reviewer’s comments and revision of the manuscript. Here we summarized the changes from the previous version of our manuscript.

**Requests from the editors:**

1. Please carefully apply the clarifications as requested by the reviewers. Also please make sure that the code is available for review.

**Reply to Request 1:**

We present the referees’ comments in black and provide our responses to those comments in red.

Regarding the code availabile for review, we have sent an invitation to access the GitHub repository, including analysis code and metadata, model weights, and example results, to the journal email address ([ploscompbiol@plos.org](mailto:ploscompbiol@plos.org)) following the Code Availability Policy (<https://journals.plos.org/ploscompbiol/s/code-availability>) at the first submission. We are worried about the possibility that the information has not been shared with the reviewers yet. We kindly request that the editorial office facilitates this process. If, for some reason, it is difficult to share code privately, please let us know, and we will be happy to share it with you in public status.

**Reviewer #1:**

In their manuscript, “Network feature-based phenotyping of leaf venation robustly reconstructs the latent space”, Iwamasa and Noshita present an analysis pipeline, from image acquisition to graph-based analysis techniques, to morphometrically analyze leaf venation in five angiosperm species.

I very much enjoyed this manuscript and I believe that these approaches are needed in the leaf venation community. As the authors state, the current state-of-the-art is mostly focused on applying statistics to simple parametric measurements (like length, width, numbers of loops, etc) and the use of CNNs and other modern approaches are desperately needed for segmentation. This manuscript addresses all these issues together and I believe it will have a big impact.

**Reply to Reviewer summary:**

Thank you very much for your thoughtful comments and suggestions, which helped us to improve the quality of our manuscript. We answered your questions and comments in the point-by-point manner below:

1. My biggest criticism: zenodo links to the github and the data repositories were redacted in the manuscript and I was not able to evaluate the code. This is going to be the most impactful element of this work, and I hope that the fully reproducible workflow, using the cleared leaf database and U-NET for image segmentation especially, is provided upon publication.

**Reply to major comment 1:**

We completely agree that the public release of data and analysis code is essential for ensuring reproducibility and enabling community validation. While I may not have the precise details of the sharing process in the editorial office, we have already sent an invitation for accessing the GitHub repository, including analysis code and the metadata of the *good-quality* cleared leaves, model weights of U-Net, and example results, to the journal email address ([ploscompbiol@plos.org](mailto:ploscompbiol@plos.org)) following the Code Availability Policy (<https://journals.plos.org/ploscompbiol/s/code-availability>) at the first submission. We are worried about the possibility that the information has not been shared with the reviewers yet.

Initially, we planned to make the untreated leaf dataset publicly available at the time of publication of the paper, but we have made it available publicly at Zenodo (https://doi.org/10.5281/zenodo.7070266).

Thus, we included the sentence regarding the dataset as follows (L136):

*“The images of the untreated leaf dataset have been publicly available at Zenodo [35].”*

Some overall thoughts:

2. -It was good to see a truthful analysis of the underestimation of edges and nodes in non-chemically treated leaves. This is to be expected, and the method is powerful because it can make use of the public cleared leaf dataset and apply to backlit photos!

**Reply to major comment 2:**

We appreciate this comment, as we shared a similar concern regarding the validity of the backlighting method in capturing details of the leaf veins.

3. -The graph-based analysis is refreshing to see. The paper focuses on the latent space, and example venation images are shown for datapoints in the PCA space. Do the authors think they could somehow derive meaningful eigen-representations of venation networks?

**Reply to major comment 3:**

The principal component (PC) scores, or a derived score (e.g., a score along a one-dimensional (1-D) U-shaped distribution pattern) can be meaningful eigen-representations of venation networks. Fig. 7A illustrates the robust reconstruction of the 1-D U-shaped distribution pattern, a latent space, in both datasets, where the network structures transformed from looping to tree-like structures along the 1-D U-shaped curve. We visually identified the biological and network structural meanings of the latent space (Fig. 7B) and quantitatively in terms of the network features (Fig. 7C).

However, we think that we have not yet achieved deriving ideal eigen-representations of venation networks which can generate examples of venation networks in any point of the morphospace. Our expectation is that such eigen-representations would enable the inverse analysis of the leaf veins to facilitate the investigations of their functionalities and simulations. We plan to undertake this as the next step.

4. -Related to the above, I wonder if a discussion of other graph-based analysis approaches, like vector representations through node embeddings or graph distances based on the segmented network could be incorporated. Deriving biological meaning from these powerful mathematical representations of graphs will be interesting to look at as more venation networks are analyzed.

**Reply to major comment 4:**

We agree that other graph-based analyses, including node embedding, graph embedding, and clustering with graph distances, are interesting and could help generate good representations of venation networks. Especially graph neural network (GNN)-based embedding and generative models might be promising techniques. Accordingly, we added the sentence in Discussion as follows (L342-345):

*“Although the proposed method cannot reconstruct vein network structures at arbitrary points in the morphospace, the integration of embedding techniques utilizing graph neural networks and generative models [45] holds promise in expanding the method toward inverse analysis throughout the entire morphospace.”*

I enjoyed this manuscript a lot, great job!

We are glad to see this encouraging comment.

**Reviewer #2:**

the review is uploaded as an attachment

Comments for Author

This manuscript reports the characterization of leaf venation patterns, emphasizing the importance of high-throughput and objective statistics using network features. It is pointed out that the conventional analyses of leaf venation were relatively simple and did not capture inherent complexity in venation patterns, therefore, the authors focused on the quantification of vein networks and found a characteristic one-dimensional axis in morphospace which may be a useful morphological descriptor suitable for classifying the leaf venation.

I find the method used in this work fascinating and the findings are interesting and informative, especially the introduction of machine-learning image analysis and the evaluation of the network features. I would say that this work merits the attention of a broad readership and worthy of publication. However, I would like to recommend further clarifications on some minor points outlined below.

**Reply to Reviewer summary:**

Thank you very much for clearly summarizing our study and sharing your insightful comments and suggestions. Please find our reply to your comments below:

Minor points:

1. In the beginning of Abstract (and Author Summary), the purpose of the study seems lacking. There are some key words such as “network pattern is shared in clades”, “functional demands constrain the network structure”, “biased distribution in the morphospace” but then what is the purpose of this study? Possibly, it might be an evolutionary process, species identification only by leaf venation, but it should be clearly stated.

**Reply to major comment 1:**

We updated the following sentence for clarifying the purpose of the study (L23-24 and L45).

L23-24

*“To better understand the morphological diversities and constraints on leaf vein networks, high-throughput phenotyping workflow for the quantification of vein networks and identified leaf venation-specific morphospace patterns.”*

L44-45

*“We used network features to characterize hierarchical and complex venation patterns for quantitatively understanding their morphological diversities and constraints.”*

2. In the end of Abstract (and Author Summary), the meanings of functional trade-offs morphological constraints were not clear. I think at least in Author Summary, it should be clearly stated for non-technical readers, e.g., trade-offs between what and what.

**Reply to major comment 2:**

Thank you for your suggestion. We added the following explanations to clarify what we consider mainly in this study (L32-35 and L49-51).

L32-35

*“…images. The one-dimensional distribution patterns suggested that venation patterns are determined by a functional trade-off because they align with the Pareto front that optimizes transport efficiency, construction cost, and robustness to damage, as predicted by the earlier theoretical study.”*

L49-51

*“The biased distribution patterns corresponded to a one-dimensional curve, which was predicted by a theoretical study based on the functional trade-offs between optimizing transport efficiency, construction cost, and robustness to damage.”*

3. I felt a bit confused about Abstract and Author Summary because the authors concluded the approach is promising for analyses of the functional and structural properties of veins in Abstract but they concluded the approach is for analyses in various other fields of research including biomimetics, generative design. It could be better to make them consistent.

**Reply to major comment 3:**

We appreciate this comment, as we initially thought it better to make consistent Abstract and Author Summary. However, the submission guidelines (https://journals.plos.org/ploscompbiol/s/submission-guidelines) said that the following are required for Author Summary:

*Aim to highlight where your work fits within a broader context; present the significance or possible implications of your work simply and objectively; and avoid the use of acronyms and complex terminology wherever possible. The goal is to make your findings accessible to a wide audience that includes both scientists and non-scientists.*

Thus, we decided to highlight where our study within a broader context for accessibility to a wide audience, including non-scientist, in Author Summary.

4. Line 65: It needs a clear explanation about “Leaf venation is under morphological constraints related to functional demands”. I think some key words for function are needed such as hydraulic transporation, transpiration, photosynthesis otherwise the readers may be lost about what the authors want to claim.

**Reply to major comment 4:**

We added the examples of functional demands as follows (L73-75):

*“Leaf venation is under morphological constraints related to functional demands (e.g., hydraulic conductivity, transpiration efficiency, and tolerance to damage and blockage)…”*

5. Line 234 underestimated: I didn’t think there may be a correlation between number of nodes and leaf area because their relationship is not so trivial. Regarding this, the five times difference is not surprising. It might be better to have an expectation or a logical connection before the explanation of Fig.5.

**Reply to major comment 5:**

Qualitatively, this correlation between the number of nodes and leaf area was to our expectations and previous studies (e.g., Sack et al., 2012). It could reflect this empirical law that certain measurements per unit area (e.g., VLA) have been employed for evaluation. On the other hand, we agree that the correlation is not simplistic. We consider that we currently lack a rational and complete explanation of it, and it ought to be addressed through further investigations from functional, evolutionary, physiological, and developmental perspectives. Here, we decided to show our measurement findings.

6. Line 257: “PC1 represented the loopiness of higher-order veins” is not trivial. It might be better to state the logical connection between PC1 and the loopiness. I admit that there are some statements in Methods part but it should be also emphasized in the main text. By the way, it should be better to define the loopiness more clearly in the main text. Same for kurtosis and PC3 in Line 273-275.

**Reply to major comment 6:**

In this study, we introduced the term “loopiness” to represent the frequency of looping structures, which correspond to cycles in graph theory, in the leaf vein network. Specifically, we hypothesized that a high degree of loopiness corresponds to a vein network with a more frequent occurrence of cycles than other networks. We updated the relevant sentence to clarify the meaning of loopiness as follows (L277-278).

*“PC1 represented the loopiness (i.e., the frequency of looping structures, known as cycles in graph theory, in a leaf vein network) of higher-order veins (Fig 7B).”*

One of the major features contributed to the PC1 score was the number of neighbors of the egonet (one-hop). The loopiness was attributed in part to the number of neighbors of the egonet (one-hop); the tree-like structures appeared when the number becomes ca. two because the neighbor nodes of an egonet show one-dimensional connections or branching structures without looping through the egonet (Fig. S4A), and the looping structures appeared when the number becomes ca. six because the neighbor nodes easily connect each other (Fig. S4B). The number of neighbors of the egonet (one-hop) show concentrated distributions (high kurtosis, low standard deviation (SD)) around two or six when the low and high loopiness, but broader distribution (low kurtosis, high SD) when the intermediate network structures including both looping and tree-like structures (Fig. 7C). We updated the relevant sentences and added supplementary figure to explain of the relationship between loopiness and PC1, and PC3 (kurtosis and SD) (L305-309, Fig. S4).

*“Our results showed that the tree-like and looping structures in the vein networks corresponded to the concentrated distributions (i.e., low standard deviation and high kurtosis) around two and six neighbors of the one-hop egonets, respectively, while intermediate structures exhibited broad distributions across both (i.e., high standard deviation and low kurtosis).”*

7. Line 262-265 “The number of … intersection nodes (Fig 7C, high PC1)”: I could not find a logical connection between the number six and intersection nodes. It is better to explain an expectation or possibilities to think about the number of neighbors in the egonet.

**Reply to major comment 7:**

We apologize for not meticulous explanation of the relationship between the number of neighbors in the egonet and intersection nodes. It is a result of our quantification, and we considered not completely explaining the relationship between them. However, it would be possible to interpret the results with more detailed logical connections. Based on the algorithms for node extraction and edge detection (Fig. 3), vein networks were represented as undirected graphs without crossing edges; consequently, the number of edges per node was concentrated around one (endpoint node; Fig. 3A) or three (intersection node; Fig. 3B). In the case of the number of edges per node being one, a one-hop egonet derived from an endpoint node with one neighbor is a subgraph including two nodes, which are the endpoint node and its neighbor. Thus, the number of neighbors of the egonet (one-hop) concentrated to two (Fig. 7C). In the case of the number of edges per node being three, a one-hop egonet is expected to possess two, four, and six neighbors. In our observation, the six neighbor nodes frequently appeared (Fig. 7C).

Thus, we updated the relevant sentences as follows (L284-295):

*“The network features related to the egonet showed a high factor loading for PC1, with the number of neighbors of the one-hop egonet significantly contributing to the local looping structures in the vein networks. Vein networks were represented as undirected graphs without crossing edges using algorithms for node extraction and edge detection (Fig. 3), resulting in a concentration of one edge per node (endpoint nodes; Fig. 3A) or three edges per node (intersection node; Fig. 3B). In the case of the number of edges per node being one, a one-hop egonet derived from an endpoint node with one neighbor is a subgraph including two nodes, which are the endpoint node and its neighbor; the number of egonet neighbors was concentrated at two for the low-looping veins with many endpoint nodes (Fig 7C Low PC1 of cleared leaf veins, Fig. S4A). For veins with three edges per node, the one-hop egonet is expected to possess two, four, or six neighbors. In our observation, the six neighbor nodes frequently appeared (Fig. 7C High PC1 of cleared leaf veins, S4B Fig.).”*

8. Line 329-332: This statement is interesting but it seems difficult to follow only from Fig. 7B. It could be better to show a schematic illustration of what the authors found about the network features that retain clade-specific morphological properties. This may enhance impact of this study.

**Reply to major comment 8:**

Thank you for the comment on the clade-specific morphological properties. We added the sentence (L366-369) and supplementary figure (S5 Fig.).

L366-369

*“Although Quercus in the cleared leaf dataset distributed a broad range of the morphospace because its sample size is larger than others, Ficus and Prunus had tree-like structures within the vein networks, and Zelkova and Morella exhibited relatively high loopiness in both datasets (S5 Fig.).”*

9. Line 332-334: This is also a very interesting discussion but it seems some logical gaps are there. It was difficult to understand “there are structural and functional constraints” only from the one-dimensional occupation pattern. I guessed the network structures are systematically different along the one-dimensional axis and these different structures may contain different function. It could be better to explain such a logical connection between one-dimensional pattern and structural/functional constraint.

**Reply to major comment 9:**

We agree that there are some logical gaps here. We intended to provide a summary of what is further explained in the sentences that follow. We updated the relevant sentences to clarify the subsequent functional and structural interpretations (see also **Reply to major comment 10**).

10. Line 335-338: This is a view point for the study of leaf venation pattern, and shaped this study. However, I think it was a bit insufficient to explain the finding with some technical terminology such as locally adaptive fluctuating load and one-dimensional Pareto front. I also think this point is fully discussed in the other section besides “the robust reconstruction of morphospace”. Also, up to here, the trade-off between optimizing transport efficiency, construction cost, and robustness to damage is not clear so that it might be better to explain each step by step. Lastly, it seems interesting about the consideration of “along which the network topology changes from a reticulate to a tree-like structure” with viewing fig 2 in ref 13. But the mechanism behind was not clear only from this statement. As it is a very informative discussion, it might be better to have a schematic illustration of the Fig2 in ref 13 like the comment 14 above and precisely explain what the authors want to claim.

**Reply to major comment 10:**

We appreciate for the constructive comment and valuable suggestion. We updated the relevant sentences to explain the our interpretation step by step as follows (L374-382):

*“In a previous study, Ronellenfitsch and Katifori [13] theoretically demonstrated that network topologies show a one-dimensional Pareto front, which represents a set of network topologies that cannot improve the functionalities without degrading another, as a result of a trade-off between optimizing transport efficiency, construction cost, and robustness to damage. These functional parameters are considered to correspond to essential functional demands of leaf vein networks, such as hydraulic conductivity, transpiration efficiency, and tolerance to damage and blockage in veins [6–8]. Furthermore, they showed that the network topology changes from a reticulate to a tree-like structure along the one-dimensional Pareto front.”*

We updated Fig. 7B to show a schematic illustration similar to Fig. 2 in Ref 3.

Terms and typos:

1. Line 52:the meaning of “(characterized by) relative vein widths” was not clear.

**Reply to comment 1:**

We updated the relevant sentence to clarify the meaning as follows (L59-60):

*“Dicotyledonous leaves are characterized by hierarchical reticulate venation and relative vein widths in successive orders of the hierarchy”*

2. Line 57:“improves” hydraulic conductivity, transpiration efficiency, and tolerance to damage relative to what?

**Reply to comment 2:**

We intended that hierarchical leaf venation improves them relative to others that do not have a high hierarchy (e.g., monocots, some the eudicots). However, we were concerned that such examples would confuse, as we would like to focus on structural aspects independent of clades. Thus, we updated the relevant sentence as follows (L66-67):

*“Hierarchical leaf venation contributes to hydraulic conductivity, transpiration efficiency, and tolerance to damage and blockage in veins.”*

3. Line 66: it is better to more clearly define or introduce morphospace. The term “theoretically possible morphological spectrum” was still vague to understand morphospace.

**Reply to comment 3:**

We updated the relevant sentence as follows (L76-77):

*“… in the morphospace (i.e., in the theoretically possible morphological spectrum, wherein a venation architecture would be represented as a point within the space)”*

4. Line 88 unsuitable: In the results, it includes raw leaf samples so it might be confusing if the unsuitability is emphasized.

**Reply to comment 2:**

We appreciate this comment. We updated the relevant sentence as follows (L98-101):

*“Obtaining images of raw leaf samples with sufficient quality for extracting higher-order veins from the backgrounds can be challenging when using a digital single-lens reflex camera (DSLR) and flat-head scanner due to the low-contrast and intricate leaf textures.”*

5. Line 93: it might be “a raw leaf…system or cleaned image”

**Reply to comment 2:**

Thank you for your careful reading and helpful suggestion. We updated the sentence as follows (L105-106):

*“a leaf using a digital camera (and backlighting system for untreated leaves)”*

6. Line 99: Loopiness

**Reply to comment 6:**

We fixed the typo.

7. Line 100: It could be better to precisely state the meaning of latent data space.

**Reply to comment 7:**

We included an explanation of the meaning of the latent data space in this study as follows (L112-113):

*“… the latent data space (i.e., a low-dimensional distribution of leaf vein structures within the network feature space) …”*

8. Fig.1 caption: It could be better to define empirical morphospace. This is used for the first time here. The term is explained in the method Morphospace analysis of the leaf vein network but I could not get the meaning of “empirical”.

**Reply to comment 8:**

Thank you for the suggestion on the use of the term “empirical morphospace.” In theoretical morphology and morphometrics, empirical morphospace refers to a morphospace reconstructed *a posteriori* based on observed data, in contrast to a theoretical morphospace, which is defined *a priori* based on a theoretical morphological model. Because the first use of the word “empirical” in this caption will make confusion, we removed it here and included the explanation explicitly that the morphospace was reconstructed based on the observed data.

9. Line 170: Where is the boundary of the leaf vein?

**Reply to comment 9:**

We apologize for not meticulous explanation of the boundary of the leaf vein. We defined the boundary of the leaf vein as the leaf vein pixels of which neighbors include at least one background pixels. We updated the relevant sentences as follows (L186-187):

*“… from the boundary of the leaf vein pixels. The boundary of the leaf vein pixels was defined as the leaf vein pixels of which neighbors include at least one background pixel.”*

10. Line 216: It might be better to state the machine spec information in the method section.

**Reply to comment 10:**

Thank you for the suggestion. We added the sentence of the machine spec information to **Materials and Methods** as follows (L175-178):

*“In this study, the segmentation and following steps, including skeletonization, graph representation (****Undirected graph representation of leaf vein networks****), and network feature calculation (****Leaf vein network feature extraction****), were conducted using an Intel(R) Core i9-10900K CPU @ 3.70GHz and an NVIDIA RTX3090 GPU.”*

11. Line 228: I could not get the meaning of “generating one pixel”.

**Reply to comment 11:**

We updated the relevant sentence to explain the skeletonization step as follows (L246-248):

*“Skeletonization from the vein images successfully yielded a one-pixel representation of the vein structure while preserving the connectivity and loops.”*

12. Line 248: empirical → data-driven? I want to know the meaning of empirical. Are there any different types of morphospace besides the empirical one.

**Reply to comment 12:**

We apologize for the insufficient explanation of empirical morphospace. We adopted here the term of empirical morphospace to clarify that we reconstructed the morphospace by using PCA from the network features (see also **Reply to comment 8**). We updated the relevant sentence as follows (L221-223):

*“… in an empirical morphospace, that is, the parameter space reconstructed based on the observed data, in which each point corresponds to the structure of each venation.”*

13. Line 257: I could not get the meaning of “opposite orders for the correspondence of the PC1 axis”

**Reply to comment 13:**

We updated the relevant sentence to clarify the meaning as follows (L278-281):

*“However, opposite orders for the correspondence of the PC1 axis to the degree of loopiness were observed for untreated and cleared leaf datasets; higher PC1 results in lower loopiness for the untreated leaf dataset; conversely, higher PC1 results in higher loopiness for cleared leaf dataset.”*

14. Line 335: Theoretically

**Reply to comment 14:**

We fixed the missing authors’ names.
