# Supplementary material for: Network feature-based phenotyping of leaf venation robustly reconstructs the latent space
Source: PLoS Comput Biol. 2023 Jul 20;19(7):e1010581. doi: 10.1371/journal.pcbi.1010581 (PMC10358950; doi:10.1371/journal.pcbi.1010581)
Supplement: Attachment — Submitted filename: PBP.docx [file pcbi.1010581.s008.docx]

**Response to the reviewers’ comments**

Kohei Iwamasa, Koji Noshita

June 10, 2023

We would like to thank the reviewers for their though reading of the manuscript and their thoughtful comments and suggestions. In this letter, we present the reviewers’ comments in black and provide our responses in red. All changes made in addressing the reviewers’ comments have been incorporated in the revised version of the manuscript. All coauthors agree with this correction.

**Requests from the editors:**

1. One of the reviewers asks for some final points to be clarified. Also include the Github link to the source code in a revised version of your manuscript.

**Reply to Request 1:**

We included the sentence regarding the publicly available GitHub link and DOI issued via Zenodo (L180-181).

*“The analysis code and model weights have been publicly available at Zenodo [40] and GitHub (https://github.com/MorphometricsGroup/iwamasa-2022).”*

**Reviewer #1:**

The authors have addressed all of my concerns, and the final published manuscript should contain a publicly available github link to the code.

**Reply to comment:**

Thank you very much for your thoughtful comments. We included the sentence regarding the publicly available GitHub link and DOI issued via Zenodo (L180-181).

*“The analysis code and model weights have been publicly available at Zenodo [40] and GitHub (https://github.com/MorphometricsGroup/iwamasa-2022).”*

**Reviewer #2:**

The authors have addressed my comments in a satisfactory manner.
Now, I can clearly understand the contents and claims especially those in Fig. 7B and C.
Here are some thoughts after reading the revised version. These are very minor comments.

**Reply to Reviewer summary:**

Thank you for your thorough review of our manuscript. Please find our reply to your comments below:

1. L33 a functional trade-off between what and what might be vague by the first reading.

**Reply to comment 1:**

We updated the relevant sentence to clarify the meaning of the functional trade-off as follows (L32-35):

*“Because the one-dimensional distribution patterns align with the Pareto front that optimizes transport efficiency, construction cost, and robustness to damage, as predicted by the earlier theoretical study, our findings suggested that venation patterns are determined by a functional trade-off.”*

2. L278:"loopiness (i.e., the frequency of looping structures, ...)" spatial scale for the frequency might be vague by the first reading.

**Reply to comment 2:**

We updated the relevant sentence to clarify the meaning as follows (L279):

*“loopiness (i.e., the relative frequency of looping structures, …)”*
